# A Map-Based Service Supporting Different Types of Geographic Knowledge for the Public

**DOI:** 10.1371/journal.pone.0152881

**Published:** 2016-04-05

**Authors:** Mengjie Zhou, Rui Wang, Jing Tian, Ning Ye, Shumin Mai

**Affiliations:** 1 School of Resource and Environment Sciences, Wuhan University, 129 LuoYu Road, Wuhan, 430072, P. R. China; 2 Key Laboratory of Geographic Information System, Ministry of Education, Wuhan University, 129 Luoyu Road, Wuhan 430079, China; 3 Key Laboratory of Digital Mapping and Land information Application Engineering, National Administration of Surveying, Mapping and Geoinformation, Wuhan University, 129 Luoyu Road, Wuhan 430079, China; Kenya Medical Research Institute - Wellcome Trust Research Programme, KENYA

## Abstract

The internet enables the rapid and easy creation, storage, and transfer of knowledge; however, services that transfer geographic knowledge and facilitate the public understanding of geographic knowledge are still underdeveloped to date. Existing online maps (or atlases) can support limited types of geographic knowledge. In this study, we propose a framework for map-based services to represent and transfer different types of geographic knowledge to the public. A map-based service provides tools to ensure the effective transfer of geographic knowledge. We discuss the types of geographic knowledge that should be represented and transferred to the public, and we propose guidelines and a method to represent various types of knowledge through a map-based service. To facilitate the effective transfer of geographic knowledge, tools such as auxiliary background knowledge and auxiliary map-reading tools are provided through interactions with maps. An experiment conducted to illustrate our idea and to evaluate the usefulness of the map-based service is described; the results demonstrate that the map-based service is useful for transferring different types of geographic knowledge.

## Introduction

Geography is part of everyday life, including such aspects as the land, weather, economy, and culture. Studying geography enables people to understand our planet and its systems and to make wise decisions about the planet and its resources. The introduction and diffusion of geospatial technologies has been one of the main innovations in geography education in the past few decades. Geospatial technologies that offer access to geospatial information via digital representations (such as digital maps) and tools for interacting with those representations can be used to raise students’ understanding of local and global issues and to train important skills [1, 2].

Moreover, the digital revolution has transformed the creation, storage and transfer of knowledge [3]. Geospatial technologies should be regarded not only as a carrier for spatial information but also a type of service to transfer knowledge and facilitate the understanding of knowledge. The internet functions as a new information transfer medium and offers a variety of ways to communicate geo-information via the adequate geo-visualization of basic geo-data (topographic and thematic) [4]. The geospatial technologies used for transferring geographic knowledge to the public should be easily available and less formal than those used for geography teaching. As an omnipresent, easy-to-use tool, the web browser is the most common web-based platform that is available for public use.

One of the main goals of geography education for the public is the transfer of geographic knowledge. For the effective communication of geographic knowledge, several issues should be considered, such as which types of knowledge should be represented and how these types of knowledge should be represented. The issue concerning the types of knowledge that should be represented refers to the knowledge representation content, and the issue concerning how to represent knowledge refers to types of visualization (representation). Communication of geographic knowledge can learn from knowledge management, which refers to identifying and leveraging the collective knowledge in an organization to help the organization compete [5], and from knowledge visualization, which examines the use of visual representations to improve the transfer and creation of knowledge between at least two persons [6]. Knowledge comprises various types, which include tacit, explicit, individual, social, declarative, procedural, causal, conditional, relational, and pragmatic types; the taxonomies are important for theoretical developments in a knowledge management system. Existing wiki platforms can support explicit and social knowledge; for example, Wikipedia can support declarative knowledge, and WikiHow can support procedural knowledge. Existing blog platforms can support explicit and individual knowledge. A semantic bliki system can support various types and viewpoints of knowledge [7]. In Burkhard’s [8] knowledge visualization framework, he suggested that five types of knowledge must be visualized: declarative knowledge (know-what), procedural knowledge (know-why), experimental knowledge (know-how), orientational knowledge (know-where, e.g., knowledge sources) and individual knowledge (know-who, e.g., experts). Visualization types are grouped into sketches, diagrams, images, maps, objects, interactive visualizations, and stories. In contrast to knowledge visualization, which usually concentrates on both knowledge content and knowledge source, we concentrate on knowledge content. Additionally, the knowledge represented and transferred to the public belongs to the geography domain. Hence, the knowledge types that need to be transferred are different from knowledge visualization domain, and how to represent different types of knowledge must be discussed.

In terms of geography education for the public, online maps (atlases or map-based applications) represent the most common geographic technology used by the public due to their ease of access. The internet enables the rapid and easy creation, storage, and transfer of knowledge; however, map-based services that transfer geographic knowledge and facilitate the understanding of knowledge are still underdeveloped. Existing online maps or atlases can support limited types of geographic knowledge, and they usually focus on declarative knowledge (know-what), reflecting the characteristics, patterns and changing laws of geographic phenomena [9–11]. Furthermore, little attention has been paid to related methods of representing different types of geographic knowledge and further transferring them.

This paper aims to propose a framework for map-based services to transfer different types of geographic knowledge to the public; the knowledge must be represented before the transfer. Moreover, to ensure the effective transfer of knowledge, tools facilitating an understanding of the knowledge should be provided by the map-based service. In this paper, we discuss the knowledge types that should be represented and transferred to the public, and propose a method to support different types of geographic knowledge through a map-based service.

The remainder of this paper is organized as follows. The “Knowledge Types and Their Meanings in Geography” section 2 discusses different types of geographic knowledge that must be represented and transferred to the public. In the “Geographic Representation and Transfer” section, guidelines for effective knowledge transfer are proposed. Based on these guidelines, a framework for a map-based service is proposed. In the “Example and Experiment” section, an example is presented to illustrate our design along with an experience designed to evaluate the usefulness of the map-based service. Finally, the “Conclusions” section concludes the paper and provides further refinements and directions for our current work.

## Knowledge Types and Their Meanings in Geography

This section first introduces taxonomies of knowledge and then discusses types of geographic knowledge that must be transferred to the public.

### Introduction of knowledge types

Knowledge can be divided into various types; Alavi and Leidner [12] classified knowledge into the following types:

Tacit and explicit knowledge [13] are classified from the cognitive perspective; in practice, all knowledge is a mixture of tacit and explicit elements, rather than being one or the other. Tacit knowledge is rooted in actions, experience, and involvement in a specific context; it comprises both cognitive and technical elements. This type of knowledge is difficult to transfer to another person by writing it down or verbalizing it. Explicit knowledge is articulated, codified, and communicated in symbolic form and/or natural language. Explicit knowledge can be readily transmitted to others.

Individual and social knowledge are classified from the knowledge creator’s perspective. Individual knowledge is created by and exists in the individual [13], whereas social knowledge is created by and inherent in the collective actions of a group.

Declarative knowledge, procedural knowledge, causal knowledge, conditional knowledge and relational knowledge [14] are classified from the knowledge content perspective. Declarative knowledge, also known as know-about/know-what, is knowledge about terminology and facts; it describes things, events, or processes, and their attributes [15]. Procedural knowledge, also known as know-how, is knowledge exercised in the performance of some task; it corresponds to procedures, steps, methods and algorithms. Causal knowledge, also known as know-why, is knowledge about causes and effects. Conditional knowledge, also known as know-when, is knowledge about conditions and contexts; it is described as knowing “when” (or when not) and “why” to use declarative and procedural knowledge. Relational knowledge, also known as know-with, is knowledge about relationships among concepts.

Pragmatic knowledge is classified from an organizational perspective. This type of knowledge, such as knowledge about customers, products, processes and competitors, is useful for an organization [16].

### Types of geographic knowledge to be represented and transferred to the public

Types of geographic knowledge that must be represented and transferred to the public are related to the knowledge visualization content. From the knowledge content perspective, knowledge can be classified into declarative, procedural, causal, conditional, and relational knowledge. Geographic knowledge is also a kind of knowledge. Based on the existing knowledge classification, we classified geographic knowledge into declarative, procedural, causal, conditional, and relational knowledge. Conditional knowledge does not have significant geographic meaning, so we excluded this knowledge type. Then for our framework, we distinguish four types of knowledge: declarative knowledge, procedural knowledge, causal knowledge and relational knowledge, and we discuss their meanings in geography.

Declarative knowledge (know-what/know-about) is knowledge about the spatial (or temporal) distribution and pattern of a geographic phenomenon. Other types of knowledge are structured around the topic of knowledge.

Procedural knowledge (know-how) is knowledge about procedures, methods and related tools for making a map representing a geographic phenomenon. This type of knowledge is useful for professionals, students or individuals who are interested in geographic phenomenon representation.

Causal knowledge (know-why) is knowledge about the causes of a geographic phenomenon or related factors that influence the phenomenon.

Relational knowledge (know-with) is knowledge about the relationships among geographic phenomena. This type of knowledge can be built by an ontology or a related classification by experts. Accordingly, a map structure is established based on relational knowledge.

## Geographic Representation and Transfer

To transfer the various types of knowledge effectively, service should conform to certain guidelines. Appropriate representation is the foundation of effective transfer, and tools assisting the understanding of geographic knowledge are essential. This section first discusses the basic criteria for the effective transfer to the public of different types of geographic knowledge, and describes the corresponding representation types of different knowledge types. We then discuss the design of the map, which is the core representation type in a map-based service and tools facilitating the effective transfer of geographic knowledge, which are provided through interaction. Finally, a method to realize knowledge representation and tools in a map-based service is introduced.

### Guidelines for effective knowledge transfer to the public

Our approach focuses on geographic knowledge representation and transfer for the public. We chose a map-based service to realize knowledge representation and transfer. Using the internet as the medium to transfer geographic knowledge, a map-based service can provide the public with easy access to knowledge, that enables the public access geographic information and knowledge without downloading data. In a map-based service, maps are important resources for information discovery, exploration, and illustration, and can improve the public’s understanding of geographic knowledge. Furthermore, the map-based service can be dynamic and interactive, and using a map-based service can be interesting for the public.

To transfer different types of geographic knowledge effectively, it must fulfill the following basic criteria.

1. Knowledge transfer. The service must able to depict different types of geographic knowledge, including declarative knowledge, procedural knowledge, causal knowledge, and relational knowledge.
2. Visual notation. It must make use of visual representation.
3. Knowledge background. It must be communicable in the sense that the knowledge can be communicated to others of different knowledge backgrounds.

According to criterion 1, declarative knowledge is represented, followed by procedural knowledge, causal knowledge and relational knowledge, which are structured around it. A map-based service should support these types of geographic knowledge.

According to criterion 2, different types of geographic knowledge are represented visually wherever possible, and we must determine how to represent these types of knowledge.

**Fig 1:**
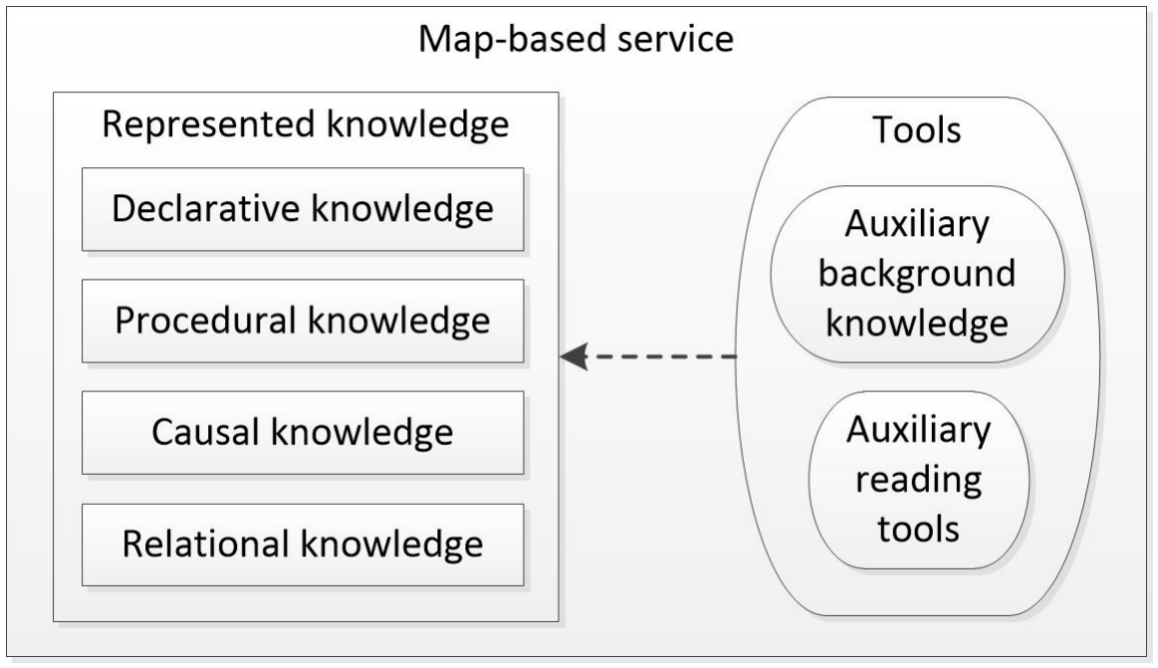
Structure of a map-based service.

According to criterion 3, it is essential to provide the public with knowledge over the web in an easily understandable, yet comprehensive manner. Due to the existence of different knowledge backgrounds, to be understood the presented knowledge must be equipped with background knowledge (here, “background knowledge” refers to information that is essential for understanding a situation or problem). Thus, tools such as auxiliary background knowledge, which provides related background knowledge to the public, and auxiliary map-reading tools, which help the public use the map-based service, should be provided to ensure that the knowledge can be communicated effectively to others. Based on the criteria described above, the structure of a map-based service is shown in Fig 1.

### Representing different types of geographic knowledge

Different types of geographic knowledge can be represented by different methods, with reference to the classification linking representation types to knowledge types summarized in Table 1. A map is the core representation type in a map-based service.

**Table 1:** Knowledge types and their corresponding representation types.

Knowledge type	Representation type
**Declarative knowledge**	Map
**Procedural knowledge**	Diagram (flowchart) or text
**Causal knowledge**	Diagram and map
**Relational knowledge**	Diagram or text and map

Declarative knowledge can be represented by a map. Declarative knowledge is the topic knowledge on the current page; other types of knowledge are structured around the topic knowledge.

Procedural knowledge can be described by text or a diagram (flowchart). For example, hyperlinks to web pages describing related procedures, methods and tools for making the map can be provided to help the user learn the procedural knowledge. Usually, only the procedural knowledge about the map that is unfamiliar to users is described.

Causal knowledge can be represented by maps and diagrams. The cause of the geographic phenomenon or related factors that influence the phenomenon can be represented by a series of maps. The structure of the cause or related influencing factors can be described abstractly and schematically by diagrams with links to the corresponding maps. When users click on the influencing factors in the diagram, the corresponding map is displayed on the page.

Relational knowledge can be described by diagrams or text, and knowledge about the geographic phenomenon that is related to the topic knowledge is represented by maps. For example, maps related to the topic knowledge can be listed on the page; when users click on the map title, a hyperlink directs them to a related map on a new page.

### Map design and tools that facilitate the effective transfer of geographic knowledge

As the main and critical type of representation of a map-based service, a map in a map-based service involves both representation and interaction. To ensure the effective transfer of geographic knowledge, the appropriate representation should be used. Moreover, static representation is not sufficient, and tools that facilitate the understanding of the knowledge represented by the map are essential. The tools include auxiliary background knowledge and auxiliary map-reading tools, and can be provided through interaction with maps. The design of map-based services has not yet been standardized, and each implementation is performed differently in terms of symbologies, interaction possibilities, functionality and usability. Nonetheless, some relevant guidelines can guide the design of online maps, such as the science of representation and interaction [17].

Map representation embraces the act of visually representing geographic phenomena in a static way. We can draw from scientific insights into visual semiotics, visual perception and visual cognition [18] to design the map representation, and we can consider graphic language [19] and map design principles [20] to ensure good cartographic design.

Cartographic interaction, which is formally defined as the dialogue between a human and a map mediated through a computing device [21], makes it possible to provide auxiliary background knowledge and auxiliary map-reading tools to facilitate the transfer of geographic knowledge to the public. Auxiliary background knowledge provides background knowledge of the represented geographic phenomenon, such as the definition and classification of a term. This type of knowledge facilitates the process of communicating geographic knowledge to users with different knowledge backgrounds. Auxiliary map-reading tools provide tools or functions to assist with map reading during the map-reading process, which includes detection, discrimination, identification and interpretation [22]. Map-based service users must be able to detect the map symbols, discriminate between different types of symbols (detection and identification), recognize the symbols as things and attach meaning to them (identification) and finally interpret information from the map and understand the knowledge transferred by the map (interpretation). Based on the different stages of the map-reading process, auxiliary map-reading tools are divided into three types: auxiliary “detection” and “discrimination" tools, auxiliary “identification” tools and auxiliary “interpretation” tools.

To implement auxiliary background knowledge and auxiliary map-reading tools, interaction primitives can be used, especially work operator primitives for map-based visualization [23]. Work operator primitives and their definitions are presented in Table 2.

**Table 2:** Work operator primitives [23].

Work operator primitive	Definition
**Reexpress**	Interactions that change the visual isomorph
**Arrange**	Interactions that manipulate the layout of views in a coordinated visualization
**Sequence**	Interactions that generate an ordered set of related maps
**Resymbolize**	Interactions that change the design parameters of a map type without changing the map type itself
**Overlay**	Interactions that adjust the feature types included in the map
**Reproject**	Interactions that change the map projection by translating coordinates on the curved Earth to a flat plane
**Pan**	Interactions that change the geographic center of the map and are used when a portion of the map is off screen
**Zoom**	Interactions that change the scale of the map
**Filter**	Interactions that identify map features that meet one or a set of user-defined conditions
**Search**	Interactions that identify a particular location or map feature of interest
**Retrieve**	Interactions that request specific details about map features of interest
**Calculate**	Interactions that derive new information about map features of interest

Auxiliary background knowledge can provide background knowledge. This tool is provided through interactions with the map features, and work operator primitive: retrieve can be used to realize it. For example, map titles and textual descriptions in the map may contain terms that users do not understand. Therefore, definitions of the terms are provided (when the cursor hovers over a term, its definition is displayed) to facilitate users’ understanding of the relevant content.

The auxiliary “detection” and “discrimination” tools can be provided to users to manipulate the user’s viewpoint of the map based on their requirements and interests; this allows computer screen size limitations to be overcome. Work operator primitives: pan and zoom can be used to realize these tools.

The auxiliary “identification” tool can help users accomplish the act of identification directly through retrieval or even acquire more information. Work operator primitive: retrieve can be used to realize this tool. For example, quantitative data in thematic maps generally must undergo a data analysis and classification process, which makes it difficult for users to obtain accurate values for statistical data. As a result, auxiliary “identification” tools can be provided to access the underlying data.

The auxiliary “interpretation” tool can help users interpret information and understand the knowledge transferred through the map, such as manipulating the type, layout, order and design of a map and examining features in the map. Work operator primitives: arrange, sequence, reproject, overlay, reexpress, resymbolize, filter, search, retrieve and calculate can be used to realize this tool. The functions and implementation of different types of tools are summarized in Table 3. Different work operator primitives can be chosen and used according to specific maps and applications.

**Table 3:** Auxiliary background knowledge and auxiliary map-reading tools.

Type	Function	Work operator primitives
**Auxiliary background knowledge**	Provide background knowledge	Retrieve
**Auxiliary map-reading tools**		
**Auxiliary “detection” and “discrimination” tools**	Help users detect and discriminate the symbols on the map	Pan, zoom
**Auxiliary “identification” tools**	Help users identify map symbols	Retrieve
**Auxiliary “interpretation” tools**	Help users interpret information and understand the knowledge transferred through the map	Reexpress, resymbolize, arrange, sequence, reproject, overlay, filter, search, retrieve and calculate

### Implementation of knowledge representation and tools in a map-based service

In this section, we introduce methods to realize the representation of knowledge and tools that facilitate understanding of the knowledge discussed above, concentrating on the methods to make maps and diagrams. Methods to establish the infrastructure of web applications are not discussed; this function can be realized through various types of technologies according to different conditions and is not the focus of this paper.

Knowledge representation and tools in a map-based service can be realized with various technologies, such as JavaScript, SVG (Scalable Vector Graphics), Flash, Java applets, and Silverlight [24]. SVG, JavaScript [11, 25], and Flash [26, 27] are currently used to publish online maps, enabling interactions with maps and map animations. SVG is easy-readable plain-text, easy-editable by a simple text editor, and can be highly compressed to reduce map or diagram size. Therefore, we chose JavaScript and SVG to publish maps and diagrams in a map-based service. The maps and diagrams are rendered as SVG graphics with the associated JavaScript interaction, which supports tools that facilitate geographic knowledge understanding. Maps are much more complex than diagrams, and maps are crucial for map-based services. We present an example map to illustrate the process of realizing knowledge representation and the tools that facilitate understanding knowledge.

**Fig 2:**
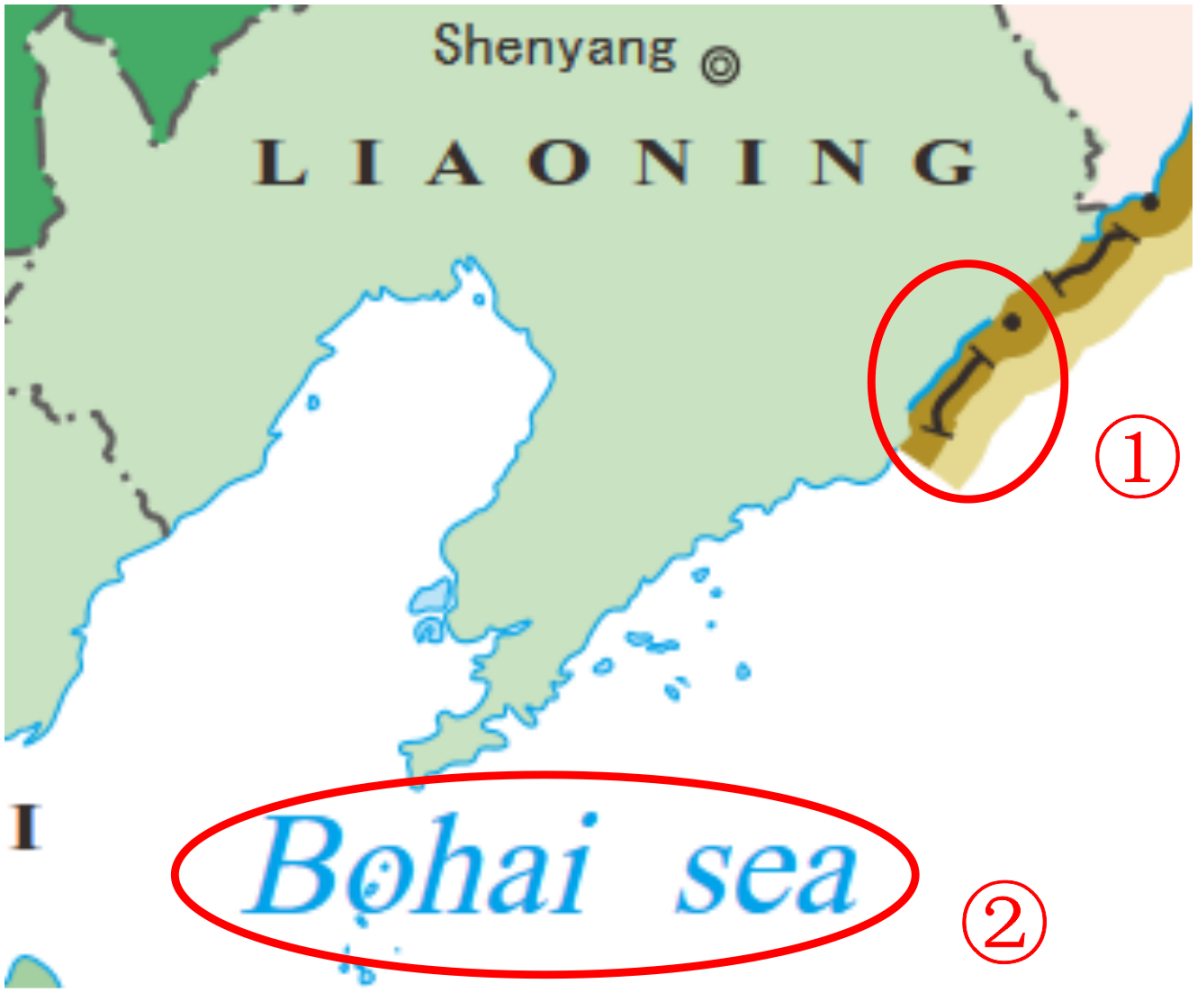
Using SVG to render a map.

1. **Using SVG to render a map.** SVG is a language for describing two-dimensional graphics in XML, which allows for three types of graphic objects: vector graphic shapes, images, and text. CSS styling can be applied to SVG content. Information in a map can be described by SVG. Map symbols can be described by vector graphic shapes in SVG, and map annotations can be described by text in SVG. As shown in Fig 2, ① the national boundaries can be described by ‘path’ element and ‘line’ elements, and ② map annotations can be described by a ‘text’ element. The ‘g’ element can group together graphic elements.
2. **Using JavaScript to add interaction or animation to a map.** JavaScript can control SVG. It can be embedded in a SVG document or linked to a SVG document. Events can cause scripts to execute if event attributes or event listeners occur. Interactions and animations, such as pan and zoom, can be added to a map through JavaScript by controlling the <viewbox> in a SVG using JavaScript, and resymbolize can be realized by controlling style elements in a SVG.

① <path fill = "none" fill-rule = "nonzero" stroke = "#332C2B" stroke-width = "83.9653" d = "M88786 34645c11,-29–5,-59–13,-87–9,-28–13,-59–9,-89l0 0c6,-44 33,-80 58,-116 26,-35 55,-68 92,-93 101,-84 144,-223 262,-290"/>
  <line fill = "none" fill-rule = "nonzero" stroke = "#332C2B" stroke-width = "41.9297" x1 = "88923" y1 = "34686" x2 = "88637" y2 = "34600" />
  <line fill = "none" fill-rule = "nonzero" stroke = "#332C2B" stroke-width = "41.9297" x1 = "89280" y1 = "34069" x2 = "89071" y2 = "33874" />
  <path fill = "#332C2B" fill-rule = "nonzero" stroke = "#332C2B" stroke-width = "26.8717" d = "M89502 33642c17,33–5,74–34,86–28,12–60,0–85,-28–25,-28–9,-77 31,-94 39,-16 72,3 88,35z"/>
② <g transform = "matrix(1 0–0.26256 1 34181.8–5166.74)">
  <text x = "59950" y = "44081" fill = "#00A2E9" font-weight = "normal" font-size = "1381.6px" font-family = " Aparajita ">Bohai</text>
  <text x = "63765" y = "44081" fill = "#00A2E9" font-weight = "normal" font-size = "1381.6px" font-family = " Aparajita ">sea</text>
  </g>

## Example and Experiment

We present an example to illustrate our idea and describe an experiment that was performed to evaluate the usefulness of the example of a map-based service. The map-based service can transfer different types of geographic knowledge to the public; here, the knowledge centers on the population distribution in China, including declarative knowledge about the distribution pattern of the population, procedural knowledge about how to create a map representing the population distribution, causal knowledge about the factors that influence the population distribution, and relational knowledge about geographic phenomena related to the population distribution.

**Fig 3:**
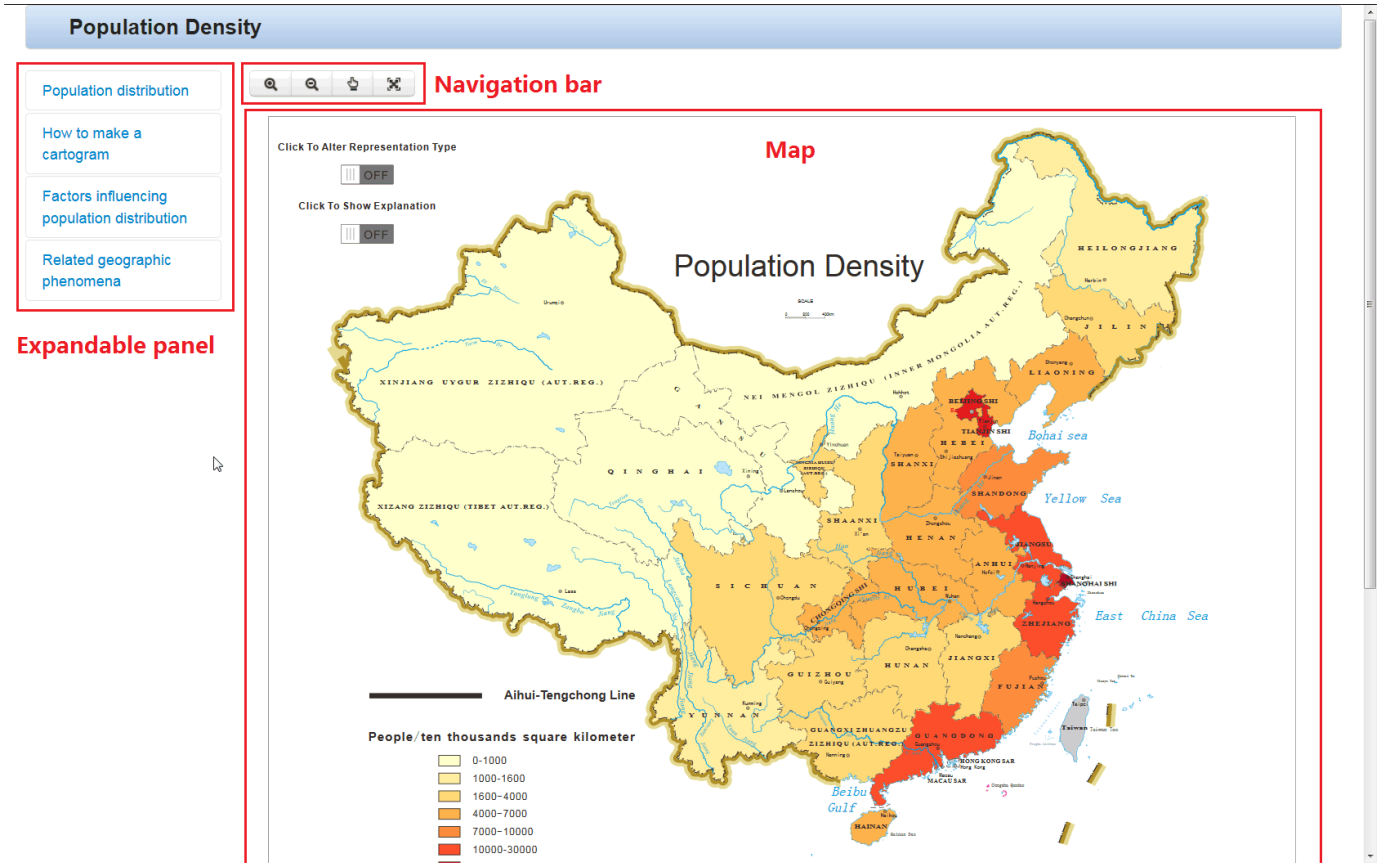
The interface of the map-based service.

The interface of the map-based service is shown in Fig 3. As shown in Fig 3, a map is displayed in the web browser window with an expandable panel and a simple navigation bar. The expandable panel on the left of the window lists the four types of knowledge with an”accordion” effect, which essentially involves the user clicking on a panel heading to reveal the content underneath, thus controlling the display of the various types of knowledge. The simple navigation bar on the top of the window provides the basic function of manipulating the viewpoint of the map displayed.

### Declarative knowledge (know-about): knowledge about the distribution pattern of the population in China

The map, which represents the knowledge, is displayed when the user clicks on the content underneath the “know-about: population distribution” panel heading, as shown in Fig 3. Auxiliary background knowledge and auxiliary map-reading tools are provided to help users understand this map.

Auxiliary map-reading tools: auxiliary “detection” and “discrimination” tools (work operator primitive: zoom and pan). Users’ viewpoints on the map can be manipulated based on their requirements and interests through interactions with the navigation bar and map.

Auxiliary “identification” tools (work operator primitive: retrieve): When the cursor hovers over a province, an accurate attribute value of the appropriate population density is displayed.

**Fig 4:**
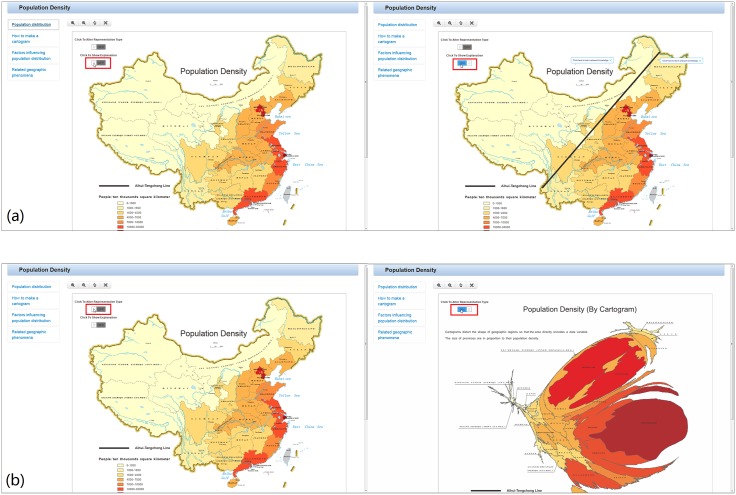
Declarative knowledge: population distribution represented by an interactive map (data year: 2013, data source: the China statistical Yearbook. http://www.stats.gov.cn/tjsj/ndsj/2014/indexch.htm). (a) Auxiliary “interpretation” tools (work operator primitive: overlay) show an explanation of the map. (b) Auxiliary “interpretation” tools (work operator primitive: reexpress) provide an interesting representation.

Auxiliary “interpretation” tools (work operator primitive: overlay and reexpress): The geo-demographic demarcation line, the Aihui-Tengchong line, is overlaid through interactions with a button on the map labeled “click to show explanation”, as shown in Fig 4a. A “distorted map”, cartogram, is used to reexpress the population density in China. The cartogram distorts the shape of a geographic region so that the area directly encodes a data variable and provides an interesting view to users who “turn on” a switch on the map labeled “click to alter representation type”, as shown in Fig 4b.

Auxiliary background knowledge (work operator primitive: retrieve): When users click on the map title, a hyperlink directs them to a Wikipedia article about “population density”. When users click on the Aihui-Tengchong line, a hyperlink directs them to a Wikipedia article about the “Aihui-Tengchong line”. Auxiliary background knowledge helps users better understand the map by providing access to relevant knowledge, which ultimately improves the transfer of knowledge.

### Procedural knowledge (know-how): knowledge about how to create a cartogram

This type of knowledge is displayed through a hyperlink to a web page describing the procedure to create a cartogram, which is accessed when the user clicks on the content underneath the “know-how” panel heading.

### Causal knowledge (know-why): knowledge about factors that affect the population distribution in China

**Fig 5:**
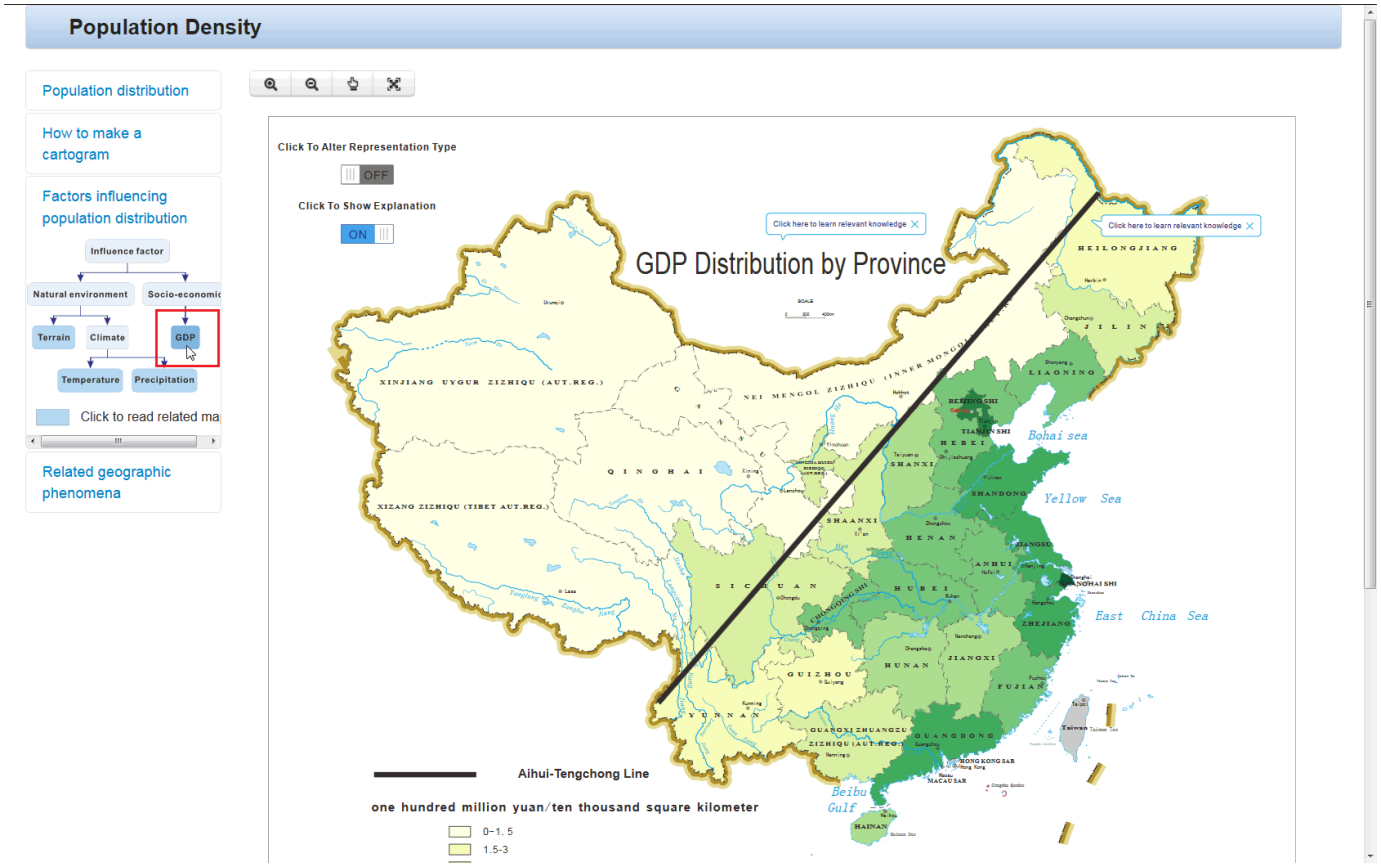
Causal knowledge: one of the factors that affect the population distribution in China. Factors influencing population distribution: socio-economic—GDP (data year: 2013, data source: the China statistical Yearbook. http://www.stats.gov.cn/tjsj/ndsj/2014/indexch.htm).

The influencing factors are represented abstractly by a node-link diagram underneath the “know-why” panel heading, and the leaf node can be an indicator that represents the corresponding influencing factor to some extent. Factors influencing population distribution can be divided into natural environment factor and socio-economic factor. Natural environment includes terrain and climate (temperature and precipitation), and socio-economic factor includes GDP. When users click on the leaf node in the left diagram, the relevant map is displayed on the right of the page, as shown in Fig 5.

### Relational knowledge (know-with)

**Fig 6:**
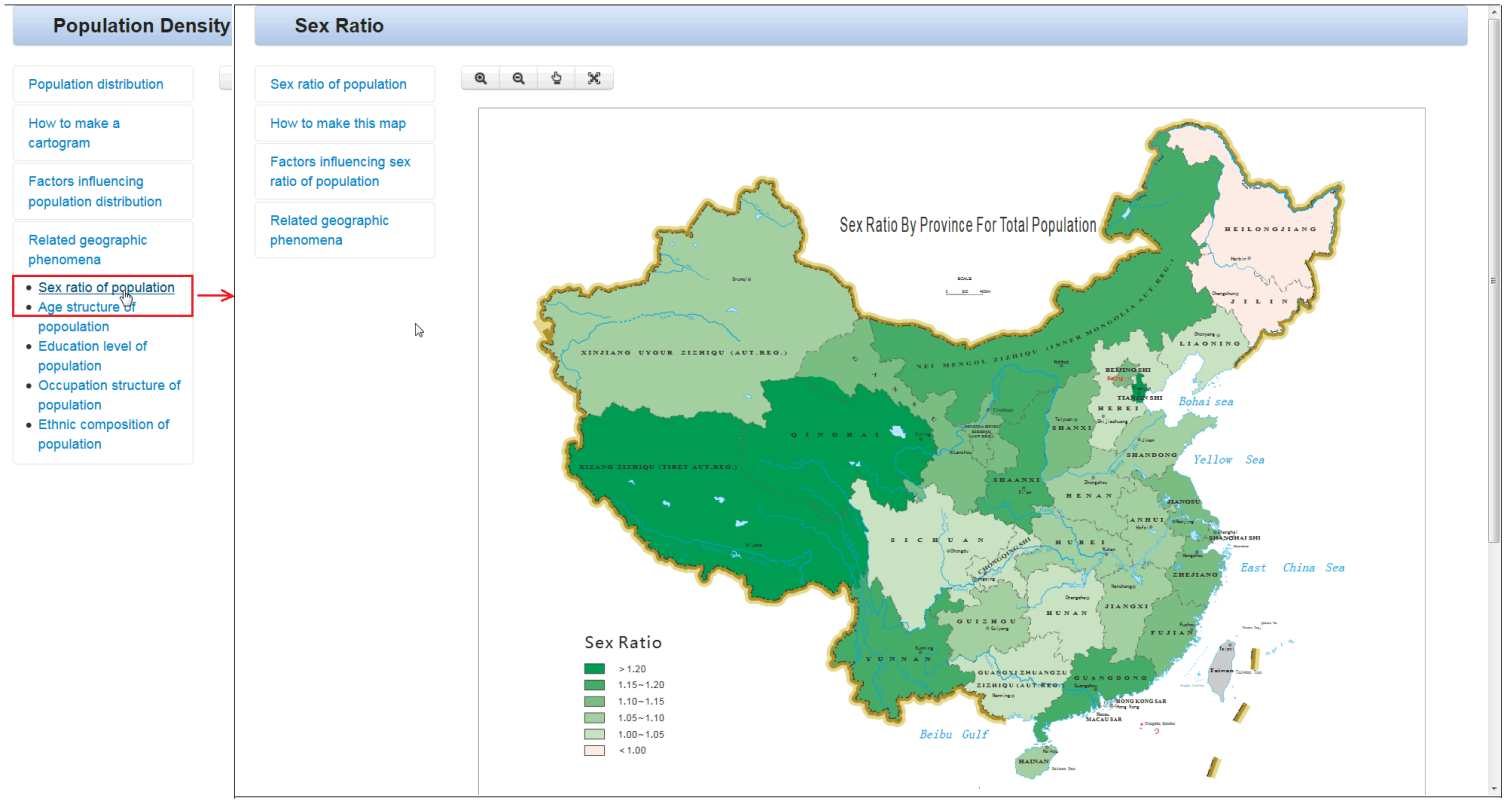
Relational knowledge: related geographic phenomena (data year: 2013, data source: the China statistical Yearbook. http://www.stats.gov.cn/tjsj/ndsj/2014/indexch.htm).

Relationships exist among geographic phenomena, so related geographic phenomena are provided. As recommended national standards in China, classifications and codes for thematic map information (GB/T18317-2009) are used to build the knowledge in this experiment. Hyperlinks directing users to related geographic phenomena, including the sex ratio, age structure, education level, occupation structure and ethnic composition of the population, are provided underneath the “know-with” panel heading. As shown in Fig 6, when users click on the sex ratio of the population, the related map is shown.

We made comparisons with Maplet, an online map platform for teaching, learning, and popularization of science (http://maplet.osgeo.cn/). In the terms of the supported geographic knowledge types, Maplet can only support declarative knowledge, while our map-based service can support declarative knowledge, procedural knowledge, causal knowledge, and relational knowledge. In terms of representation types, Maplet used maps and text, while our map-based service used maps, diagrams, and text. In terms of tools that facilitate the effective transfer of geographic knowledge, Maplet only allowed for basic cartographic interaction including zoom and pan, while our map-based service can provide Auxiliary background knowledge and auxiliary map-reading tools (such as reexpress, arrange, sequence, resymbolize, overlay, pan, zoom, and retrieve).

To evaluate the usability of map-based service, we conducted a user survey with 62 student participants. These students came from different universities in Wuhan with different educational backgrounds and were randomly divided into two equal size groups. The first group was asked to use the map-based service; the second group was asked to use a handout that was designed to transfer the same geographic knowledge as the map-based service. Students in the two groups were asked to learn for 30 minutes and then take a test (see S1 Appendix. Question paper) to answer the related questions about the geographic knowledge transferred by the map-based service or handout. These questions were divided into four parts: A for testing declarative knowledge, B for testing procedural knowledge, C for testing causal knowledge, and D for testing relational knowledge. We expected no significant differences between the scores of the two groups. Table 4 shows the scores of each group.

**Table 4:** Scores of the two groups.

Group	Score of part A	Score of part B	Score of part C	Score of part D
**Group 1**	25, 24, 23, 21, 20, 19, 26, 23, 23, 24, 25, 25, 26, 21, 23, 24, 22,22, 23, 24, 23, 22, 22, 23, 25, 23, 24, 24, 24, 20, 23	19, 18, 18, 16, 19, 19, 20, 17, 17, 18, 19, 13, 20, 15, 18, 17, 16, 16, 17, 17, 17, 17, 16, 19, 17, 18, 18, 16, 14, 14, 17	20, 22, 18, 19, 17, 21, 24, 21, 22, 22, 23, 23, 19, 21, 21, 20, 21, 20, 21, 21, 19, 20, 23, 21, 21, 22, 22, 22, 18, 21, 19	24, 20, 18, 19, 18, 17, 23, 20, 20, 22, 21, 22, 22, 19, 20, 21, 19, 19, 21, 20, 19, 20, 18, 22, 21, 20, 22, 21, 16, 21, 20
**Group 2**	24, 22, 24, 20, 22, 23, 19, 20, 20, 21, 20, 21, 23, 22, 18, 21, 22, 22, 21, 22, 23, 23, 22, 22, 22, 21, 21, 21, 18, 19, 20	19, 19, 16, 20, 17, 18, 13, 15, 14, 16, 17, 17, 18, 13, 17, 14, 16, 17, 16, 17, 18, 19, 17, 17, 17, 16, 16, 15, 13, 15, 17	15, 18, 21, 17, 16, 17, 14, 15, 16, 15, 16, 16, 17, 16, 15, 15, 14, 20, 17, 18, 19, 16, 16, 17, 16, 17, 16, 15, 13, 19, 20	22, 21, 24, 16, 19, 20, 16, 18, 17, 18, 19, 20, 19, 19, 17, 18, 17, 19, 19, 20, 20, 22, 19, 20, 19, 23, 18, 18, 17, 18, 19

On average, the scores of group 1 were higher than that of group 2(as shown in Table 5). The results of the two-sample t-test Table 6) show that the scores of the two groups were significantly different in each part, except for in part B. The results show that the map-based service was useful. The methods to support procedural knowledge need to be redesigned to improve their ability to transfer knowledge. For example, we could use a flowchart to visualize procedural knowledge rather than a verbal description of the procedure accessed via a hyperlink, and we could show the flowchart on the current page to reduce the need to transfer between pages.

**Table 5:** Group statistics.

	n_1_	n_2_	${\overset{-}{x}}_{1}$	${\overset{-}{x}}_{2}$	s_*1*_	*s*_*2*_
**Part A**	31	31	23.10	21.26	1.700	1.548
**Part B**	31	31	17.16	16.42	1.695	1.803
**Part C**	31	31	20.90	16.52	1.640	1.860
**Part D**	31	31	20.16	19.06	1.753	1.896

**Table 6:** Results of two sample *t*-test on scores of two groups of participants.

	Levene’s Test for Equality of Variances		t-test for Equality of Means				
	F	P-value	t	df	P-value (2-tailed)	95%Condidence Interval of the Difference	
						Lower	Upper
**Part A**							
Equal variances assumed	0.024	0.878	4.452	60	0.00004	1.013	2.665
Equal variances not assumed	0.024	0.878	4.452	59.484	0.00004	1.012	2.665
**Part B**							
Equal variances assumed	0.211	0.647	1.669	60	0.100	-.147	1.631
Equal variances not assumed	0.211	0.647	1.669	59.772	0.100	-.147	1.631
**Part C**							
Equal variances assumed	0.558	0.458	9.851	60	0.000	3.496	5.278
Equal variances not assumed	0.558	0.458	9.851	59.079	0.000	3.496	5.278
**Part D**							
Equal variances assumed	0.009	0.923	2.365	60	0.021	0.169	2.025
Equal variances not assumed	0.009	0.923	2.365	59.634	0.021	0.169	2.025

**Fig 7:**
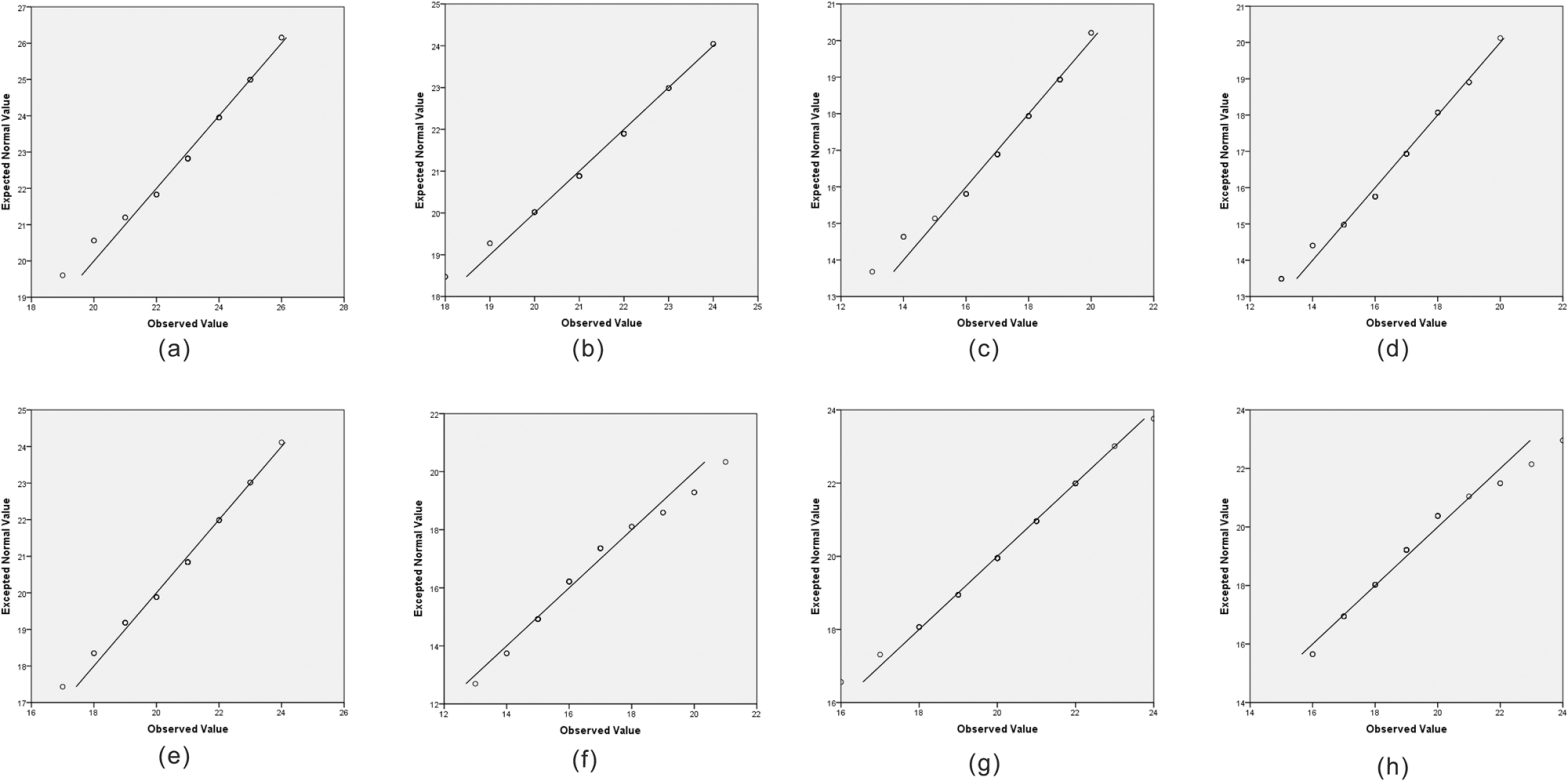
Normal Q-Q plot of the scores of the two group. (a) Part A-group1; (b) Part A-group 2;(c)Part B-group 1;(d)Part B- group 2;(e)Part C-group 1;(f)Part C-group 2;(g) Part D-group 1; (h) Part D-group 2.

1. Reasons for using the two sample *t*-test:
  Groups 1 and 2 are independent, and their sizes are small. Since the points of each Q-Q plot (Fig 7) lie close to their respective diagonal lines, we concluded that each of the group data is from an appropriately normally distributed population. Score variances in either group are unknown.
2. Hypotheses of the two sample *t*-test:
  H_0_: *μ1* = *μ2*, Null hypothesis: There is no difference between the scores of groups 1 and 2.
  Hα: *μ1*≠*μ2*, Alternative hypothesis: There is a difference between the scores of groups 1 and 2.
3. Meaning of variables used in the two sample t-test:
  ${\overset{-}{x}}_{1}$: mean of the group 1’s score
  ${\overset{-}{x}}_{2}$: mean of the group 2’s score
  *s*_*1*_: Standard deviation of group 1’s scores
  *s*_*2*_: Standard deviation of group 2’s scores
  F: Levene’s Test F value
  P-value (in Levene’s Test): the significance of Levene's test
  t: independent samples t-value
  df: degrees of freedom
  P-value (2-sided) (in two sample t-test): Probability (two-sided) that the null hypothesis is true
4. Analysis of the result of two sample t-test:
  The p values of Levene's test of each part are 0.878, 0.647, 0.458 and 0.923; they are greater than the α level (0.05). We assumed that the variances of the two groups are equal. So, we used the row labeled "Equal variances assumed." The P-values (2-tailed) of the two sample t-test in parts A, C, and D are all less than the α level (0.05), so we rejected H_0_. There is a difference between the scores of groups 1 and 2 in parts A, C, and D. The P-values (2-tailed) of the two sample t-test in part B is greater than the α level (0.05), so we failed to reject H_0_.

## Conclusions

Geospatial technologies are useful for geography education. For the general public, web browsers are the most common web-based platform available for public use, and online map-based services can be a good means of acquiring geographic knowledge. For the effective transfer of geographic knowledge, this study proposes a framework for map-based services to transfer different types of geographic knowledge to the public. The map-based service includes knowledge representation and tools facilitating the effective transfer of knowledge. We discuss types of geographic knowledge that should be represented and transferred to the public, and we propose guidelines and a method to represent different types of geographic knowledge through a map-based service. Furthermore, we illustrate our idea with the example of a simple map-based service, and we describe an experiment conducted to evaluate the usefulness of the map-based service.

To support different types of geographic knowledge, we propose a classification that links representation types to knowledge types, with maps being the core representation type in a map-based service. Tools to facilitate the understanding of the knowledge represented by the map are provided, including auxiliary background knowledge and auxiliary map-reading tools. These tools are realized through interactions with the maps in the map-based service. Moreover, methods to realize the representation of knowledge and tools to facilitate the understanding of knowledge are introduced.

We built a pilot map-based service, and we obtained positive results from a user survey on the usefulness of the map-based service. Our study shows that this map-based service is useful for transferring different types of geographic knowledge.

Although we discuss different types of geographic knowledge that must be represented and transferred, taxonomies of knowledge types are hard to define; thus, various types of knowledge supported by a map-based service are defined from specific viewpoints. A map-based service can considered to support other types of knowledge from different viewpoints, such as individual knowledge (different people may have different opinions of the spatial distribution and pattern of a certain geographic phenomenon, and a map-based service can transfer the different types of knowledge created by different individuals).

## Supporting Information

S1 Appendix Question paper. (DOCX)

S1 Video The example of a map-based service. (ZIP)
