# Supplementary material for: A Map-Based Service Supporting Different Types of Geographic Knowledge for the Public
Source: PLoS One. 2016 Apr 5;11(4):e0152881. doi: 10.1371/journal.pone.0152881 (PMC4821494; doi:10.1371/journal.pone.0152881)
Supplement: S1 Appendix — (DOCX) [file pone.0152881.s001.docx]

**S1 Appendix. Question paper**

**Part A**

1. Among the provinces listed below, which province has the lowest population density? ____

A. Jiangsu B. Hubei C. Guangdong D. Qinghai

1. Among the regions listed below, which region has the highest population density? ____
2. Northwest region B. East coast region C. Qinghai-Xizang (Tibet) Region D. Southwest region
3. Describe the population distribution pattern in China.
4. What is the “geo-demographic demarcation line” of China proposed by Chinese population geographer Hu Huanyong. Describe the meaning of this line.
5. How to understand the cartogram.

**Part B**

Give you a China province shapefile that comes with 2014 population data. Describe the procedure to make a cartogram representing the 2014 population in China.

**Part C**

Describe and analyze the factors influencing population distribution in China

**Part D**

Describe the basic characteristic of age structure of the Chinese population.

Describe the basic characteristic of sex structure of the Chinese population.

Describe the basic characteristic of occupation structure of the Chinese population.

Describe the basic characteristic of education level of the Chinese population

Describe the basic characteristic of ethnic distribution in China.
